# Risk of reinfection after two‐ or multiple‐stage knee revision surgery using superficial vancomycin coating and conventional spacers

**DOI:** 10.1002/jor.24892

**Published:** 2020-11-04

**Authors:** Florian Amerstorfer, Martina Schober, Thomas Valentin, Sebastian Klim, Andreas Leithner, Stefan Fischerauer, Mathias Glehr

**Affiliations:** ^1^ Department of Orthopedics and Trauma Medical University of Graz Graz Austria; ^2^ Department of Orthopedics and Trauma Hospital St. Josef Braunau Braunau am Inn Austria; ^3^ Section of Infectious Diseases and Tropical Medicine, Department of Internal Medicine Medical University of Graz Graz Austria

**Keywords:** coating, failed knee arthroplasty, spacer, two‐stage revision, vancomycin

## Abstract

This study investigates the effect of superficial vancomycin coating (SVC) in two‐ or more‐stage exchange procedures of prosthetic knee joint infections. We hypothesized that spacer treatment with SVC result in lower reinfection rates than conventional spacers after prosthetic reimplantation. Our secondary aim was to determine the demographic and treatment factors associated with reinfection rates. This retrospective cohort study compromised 96 cases with prosthetic knee infections. Twenty‐four cases were treated with a temporary SVC spacer and 72 cases with conventional spacers. Prosthetic reinfection occurred after a median observation period of 1.7 ± 4.0 years in 24 cases (25%). The prevalence of having a reinfection was not significantly different between the two treatment groups (13% [3 cases] in the SVC group vs. 29% [21 cases] in the conventional spacer group [*p* = .104]). In seven cases (7.3%), two in the SVC group (8.3%) and five (6.9%) in the conventional spacer group (*p* ≥ .999), histological, respectively microbiological evaluations from the intraoperative specimens revealed persistent infection at the second stage. Nevertheless, in all seven cases no significant higher risk of periprosthetic reinfection was observed during follow‐up (*p* = .750). Our secondary investigation of cofactors revealed that spacers additionally stabilized by nails were significantly associated with a 3.9‐fold higher hazard ratio of sustaining a reinfection of revision prosthesis (*p* = .005).

## 1 INTRODUCTION

Prosthetic joint infection (PJI) is a rare but serious complication after total knee arthroplasty.^1^, ^2^, ^3^, ^4^ Depending on the type of infection, different surgical strategies have been described in the literature.^5^, ^6^, ^7^, ^8^ A two stage‐exchange protocol is the preferred procedure for treating chronic infections with extensive damage of periarticular soft tissues or difficult to treat microorganisms.^5^, ^9^, ^10^ The procedure at Stage 1 includes the total removal of prosthesis and cement, extensive debridement of deficient soft tissue and avital bone, and the insertion of a temporary antibiotic‐loaded bone cement (ALBC) spacer. The ALBC is supplied as a two‐component system. The two‐components, one powder (polymethylmethacrylate [PMMA]) and one liquid monomer (methylmethacrylate) are mixed and polymerization is started by an activator (dimethyl‐para‐toluidine).^11^ Usually, the spacers are augmented with glycopeptides or in combination with aminoglycosides and act as a local antibiotic delivery device during the following 2–8 weeks.^12^, ^13^, ^14^, ^15^ One major complication during the spacer interval is a persistent infection, which may lead to a spacer exchange and a multiple‐stage procedure. To reduce the risk of a persistent infection, a combination of aminoglycoside antibiotics like gentamicin with the glycopeptide vancomycin may be used, showing synergetic effects. Hence, this antibiotic combination shows higher elution rates from PMMA spacers than when added in the same concentration alone.^16^ Furthermore, to enhance the local antibiotic effect, a new surgical technique has been established by pressing 2 g of vancomycin powder manually onto the cement surface (superficial vancomycin coating [SVC]).^17^ Augmented cement spacers release antibiotics from the spacer surface and in relation to their water absorption properties.^18^, ^19^

In a previous longitudinal case series, this technique resulted in favorable exceptional high local vancomycin concentrations without risks of systemic side effects.^17^ Besides ototoxicity, nephrotoxicity was reported as a systemic side effect, especially when vancomycin is administered together with aminoglycosides.^20^ Adverse effects have been indirectly described as an increase in serum creatinine of greater than 0.5 mg/dL or greater than 50% over baseline levels.^21^

During the spacer period, another important function of the bone cement spacer is to prevent soft tissue retraction. High mechanical spacer stability is important to avoid complications, such as loosening, dislocation and fracturing until the revision prosthesis can be implanted at Stage 2.

In this follow‐up study, we aimed to investigate the effect of SVC‐spacers in comparison to conventional spacers. Our primary aim was to investigate the reinfection rate after septic revision endoprosthesis in two‐ or multiple‐staged procedures. We hypothesized that treatment of spacers with SVC will be of good compatibility and result in lower reinfection rates after reimplantation than conventional spacers. Our secondary aim was to determine further demographic and treatment factors associated with reinfection rates.

## 2 METHODS

**Figure 1:**
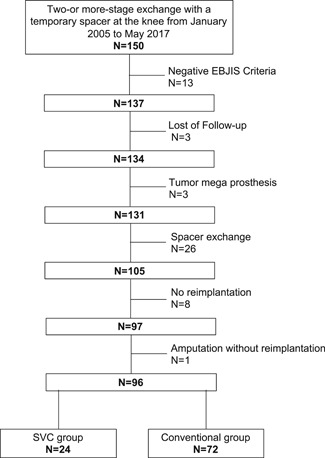
Study population: in total 150 cases were identified, where prosthesis explantation and spacer implantation was performed. After excluding 54 cases, who did not meet the inclusion criteria, 96 cases were further analyzed. EBJIS, European Bone and Joint Infection Society

This retrospective cohort study was approved by the Ethics Committee of the Medical University of Graz, Austria (EK 28‐371 ex 15/16). From January 2005 to May 2017, a two‐ or more‐stage exchange with a temporary spacer at the knee was performed in 150 cases. Inclusion criteria were cases with PJIs, where a two or more‐stage revision procedure with reimplantation of a new prosthesis was performed. Exclusion criteria applied if only spacer exchange was performed without reimplantation (*n* = 26), the proposed European Bone and Joint Infection Society (EBJIS) PJI criteria^22^ (Table 1) were not met at the time of implant removal (*n* = 13), reimplantation did not take place to prove or disprove persistent infection (*n* = 8), a mega prosthesis due to tumor resection was primarily implanted (*n* = 3), the patient was lost of follow‐up (*n* = 3) or amputation of the limb was performed instead of prosthesis reimplantation (*n* = 1). Therefore *N* = 96 cases were included in the final analysis (Figure 1). The demographics of the study population are displayed in Table 2.

**Table 1:** Proposed EBJIS criteria^22^: PJI is diagnosed if ≥1 criterion is fullfield

	**Criteria**
Clinical features	Sinus tract or visible purulence around the prosthesis
Cytology in synovial fluid	>2000/μl leukocytes or ≥70% granulocytes
Histology	Inflammation in periprosthetic tissue (>23 granulocytes per 10 HPF after Morawietz& Krenn)
Microbiology	Microbial growth in:
	Synovial fluid
	≥2 periprosthetic tissue samples*
	Sonication fluid (≥50 CFU/ml)

Abbreviations: CFU, colony‐forming unit; EBJIS, European Bone and Joint Infection Society; HPF, high‐power fields; PJI, prosthetic joint infection.

**Table 2:** Patient, operation, and microbiological characteristics

**Patient characteristics**	**Total (*N* = 96)**			**Superficial vancomycin coating (*n* = 24)**			**Conventional treatment *(n* = 72)**			***p* Value**
Age (years)	69	±11	Range: 33–88	73	±9.8	Range: 51–88	68	±10	Range: 33–87	.014
Gender										.636
Female	52	(54)		12	(50)		40	(56)		
Male	44	(46)		12	(50)		32	(44)		
ASA‐score										.797
1–2	34	(35)		9	(38)		25	(35)		
3	37	(39)		10	(42)		27	(38)		
4	25	(26)		5	(21)		20	(28)		
Diabetes	28	(29)		9	(38)		19	(26)		.300
Smoking	6	(6.3)		3	(13)		3	(4.2)		.163^a^
Immundeficiency	4	(4.2)		‐	‐		4	(5.5)		.310^a^
Operation characteristics										
Previous local revision surgeries										.699^a^
0	29	(30)		9	(38)		20	(28)		
1	53	(55)		13	(54)		40	(56)		
2	13	(14)		2	(8.3)		11	(15)		
3	1	(1.0)		‐	‐		1	(1.4)		
Spacer nail implantation	19	(20)		4	(17)		15	(21)		.774^a^
Spacer period (weeks)	11	±7.4	Range: 1.9–55	9.6	(6.6)	Range: 2.6–33	11	(7.6)	Range: 1.9–55	.180
Preoperative creatinine (*n* = 120)	1.1	±0.63	Range: 0.27–4.3	1.1	±0.60	Range: 0.41–0.32	1.1	±0.64	0.27–0.43	.867
Microbiological findings of intraoperative tissue samples										
Germ										.397^a^
Staphylococci	25	(26)		7	(29)		18	(25)		
Streptococci	11	(11)		4	(17)		7	(9.7)		
Enterococci	4	(4.2)		‐	‐		4	(5.6)		
Mixed infection	1	(1.0)		1	(4.2)		‐	‐		
Others	2	(2.1)		‐	‐		2	(2.8)		
No germ/not done	53	(55)		12	(50)		41	(57)		

Abbreviation: ASA, American Society of Anesthesiologist.

^a^ Fisher's exact test.

**Figure 2:**
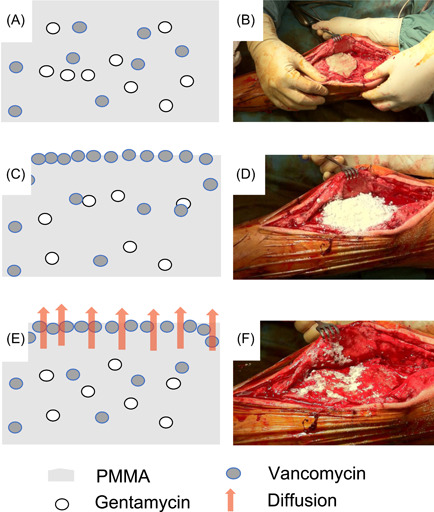
The technique of spacer preparation with superficial vancomycin coating is described graphically (A, C, and E) and documented by intraoperative pictures (B, D, and F). PMMA, polymethylmethacrylate [Color figure can be viewed at wileyonlinelibrary.com]

**Figure 3:**
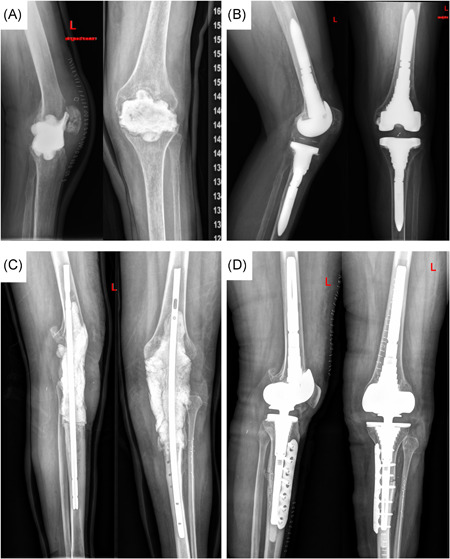
Postoperative X‐ray: (A) a static spacer without a nail. (B) At the second stage, the spacer was explanted and reimplantation was performed. (C) X‐ray of a static spacer with a nail and (D) postoperative follow‐up x‐ray, after reimplantation of a revision endoprosthesis [Color figure can be viewed at wileyonlinelibrary.com]

In the SVC group (*n* = 24) the spacer implantation was performed using the new superficial vancomycin coating (Figure 2). A compound of 80–120 g of Palacos R + G (Heraeus) was mixed with 1 g of vancomycin powder to every 40 g of bone cement to form an augmented bone cement according to the manufactural protocol. The cement was placed in the correct position to form a static spacer (Figure 2A,B). To prepare the SVC‐spacer, two additional grams of vancomycin powder was pressed manually onto the surface of the bone cement (Figure 2C–F). After hardening a wound drain was inserted but clamped for 2 h postoperatively to avoid premature washout of vancomycin. The conventional spacer (*n* = 72) was prepared in the same way, but without adding 2 g of superficial vancomycin coating. In 19 cases, a nail was inserted additionally due to the surgeon's preference or for increased stabilization after massive bone loss (Figure 3).

Daily vancomycin concentrations in wound drainage fluids and serum samples were analyzed until the drainage was removed. The analyses of the vancomycin levels were performed using a cobas c 311 Analyzer (Roche) with a detection limit of less than 2.0 μg/ml. Serum creatinine levels were analyzed preoperatively and postoperatively to indirectly detect potential nephrotoxicity. If the serum creatinine concentration increased bygreater than 0.5 mg/dl or greater than 50% above baseline level, a nephrotoxic effect was assumed.^21^

All patients received systemic intravenous antibiotic therapy during the hospital stay, followed by oral antimicrobial treatment up to the time point of their reoperation at Stage 2. The antibiotic agent was selected according to the microorganism and susceptibility. In cases of inconclusive culture, empiric antibiotic therapy was given in consultation with an infectious disease specialist. Persistent infection was defined according to the proposed EBJIS criteria (Table 1).^22^ At the time of the reimplantation of the prosthesis, intraoperative tissue samples were excised and used for further microbiological and histological investigations.

Statistical data exploration included descriptive and comparative analysis. Nonparametric bivariate analysis was performed to establish differences between the SVC and the conventional treatment, and associations between covariates and the primary outcome (re‐infection). The *χ* ^2^ test and Fisher exact test were used to detect associations between dichotomous and categorical variates, and the Wilcoxon rank‐sum (Mann–Whitney) test was used to assess relations between dichotomous and continuous variables. The Log‐rank test was used to compare two survival distributions. To evaluate independent effects of multiple predictors on the survival outcome and control for confounders, relevant variates (defined as variables with intergroup differences or with an association to the outcome at an alpha‐level of *p* < .1) were integrated into a Cox proportional hazards regression model. All evaluations were carried out with the statistical program Stata/MP 13.0 (StataCorp).

## 3 RESULTS

The study population (*N* = 96) was on average 69 years old (range: 33–88) and almost equally distributed in 52 females and 44 males. Diabetes was present in 29%, smoking in 6.3%, and immunodeficiency in 4.2%. A substantial number of patients had already undergone one or more revision surgeries in the past (70%). The isolated microorganisms in our study sample were mainly *Staphylococci* (26%) and *Streptococci* (11%). In 55% of cases, no specific pathogen could be detected preoperatively or intraoperatively at the time of spacer implantation (Table 2).

The analysis showed a significant difference in age between the SVC and the conventional group (mean difference = 5.9 years; *p* = .014). No other significant differences between the two treatment groups were identified in patient characteristics, operation, or microorganism (Table 2).

**Figure 4:**
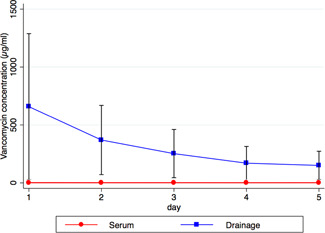
Mean (*SD*) postoperative vancomycin serum and local drainage concentrations [Color figure can be viewed at wileyonlinelibrary.com]

Figure 4 displays the measured vancomycin concentrations in blood as well as in the wound drainage over the first 5 consecutive days in the SVC group. Whereas high concentrations of local vancomycin at Day 1 (range: 74–2200 µg/ml) and a consecutive drop up to Day 5 (range: 31–407 µg/ml) occurred, serum levels remained at a low concentration at any time postoperatively (range: <2.0–10 µg/ml). The increase in serum creatinine was not different in the two treatment groups (Table 3). At the time of discharge (range: 6–22 days), five cases (5.4%) revealed a creatinine increase above 0.5 mg/dl—one in the SVC group (4.8%) and four in the conventional group (5.6%).

**Table 3:** Outcome spacer period and prosthesis

	**Total (*N* = 96)**			**Superficial vancomycin coating (*n* = 24)**			**Conventional treatment (*n* = 72)**			***p* Value**
Outcome spacer period										
Spacer infection	7	(7.3)		2	(8.3)		5	(6.9)		>.999^a^
Spacer dislocation	2	(2.1)		‐	‐		2	(2.8)		>.999^a^
Spacer fracture	1	(1.0)		1	(4.2)		‐	‐		.250^a^
Creatinine increase (*n* = 119)	−0.04	±0.38	Range: −1.7 to 1.12	−0.08	±0.36	Range: −1.3 to 0.54	−0.02	±0.39	Range: −1.7 to 1.2	.583
Creatinine increase >0.5 mg/dl (*n* = 119)	5	(5.4)		1	(4.8)		4	(5.6)		>.999^a^
Outcome prosthesis period										
Reinfection	24	(25)		3	(13)		21	(29)		.104
						RD = −0.17 95% CI (−0.34; −0.002)				
						RR = 0.43 95% CI (0.14; 1.3)				
						OR = 0.35 95% CI (0.10; 1.2)				
Follow‐up period (years; median)	2.8	±3.4	Range: 0.01–13	1.9	±3.4	Range: 0.01–4.7	3.0	±4.6	Range: 0.03–13	.040
Time of reinfection	1.7	±4.0	Range: 0.03–13	0.64	±1.1	Range: 0.35–1.4	2.2	±3.8	Range: 0.04–13	.239

Abbreviations: CI, confidence interval; OR, odds ratio; RD, risk difference; RR, risk ration.

^a^ Fisher's exact test.

The spacer interval until reimplantation was on average 11 weeks (range: 1.9–55) with no significant differences between the treatment groups. In seven cases (7.3%), two in the SVC group (8.3%) and five (6.9%) in the conventional spacer group (*p* ≥ .999), histological, respectively microbiological analysis from the specimens revealed persistent infection at the second stage. In those cases, no significant higher risk of periprosthetic reinfection was observed during follow‐up (*p* = .750). Spacer dislocation (*n* = 2) and peri‐spacer fracture (*n* = 1) showed no statistically significant difference between the treatment groups (Table 3).

**Figure 5:**
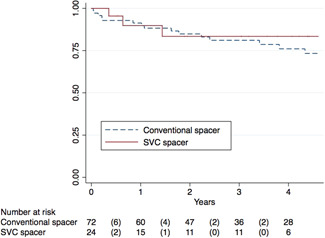
Kaplan–Meier survival estimates of revision prosthesis comparing the SVC spacer and conventional spacer group. SVC, superficial vancomycin coating [Color figure can be viewed at wileyonlinelibrary.com]

The follow‐up time of all reimplantations was in median 2.8 years (±3.4 years). The total observation period comprised 343 person‐years. In that period 24 reinfections (25%) occurred, leading to an incidence rate per person‐year of 0.07. In the observed population, 25% showed a prosthetic reinfection within 4.6 years, 50% within 10 years, and 75% within 13 years (median reinfection period = 1.7 ± 4.0 years). The prevalence of having a reinfection was 13% (3 cases) in the SVC group and 29% (21 cases) in the conventional spacer group. However, these results did not reach any statistical significance (*p* = .104) as the observation period was significantly shorter in the SVC group (1.9 ± 3.4 years) compared to the conventional control group (3.0 ± 4.6; *p* = .040) (Table 3). As a consequence, the analysis of the survival distribution revealed no significant difference between the SVC and conventional spacer group (Table 4, Figure 5).

**Table 4:** Log‐rank test for equality of survivor functions of revision prosthesis

	**Infection**		
	**Observed**	**Expected**	***p* Value**
SVC spacer	3	3.9	
Conventional spacer	21	20	
Total	24	24	.601
Spacer with nail	9	3.8	
Spacer without nail	15	20	
Total	24	24	.003

Abbreviation: SVC, superficial vancomycin coating.

**Figure 6:**
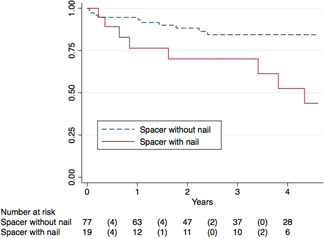
Kaplan–Meier survival estimates of revision prosthesis comparing spacer without and with nail [Color figure can be viewed at wileyonlinelibrary.com]

In contrast, spacer with nails were associated with a higher reinfection rate of the revision prosthesis during follow‐up (Table 4, Figure 6). Nineteen nails (20%) were additionally implanted with spacers; 4 in the SVC spacer group, respectively 15 in the conventional spacer group. According to the AORI classification,^23^ bone defects type I were present in 7 cases (37.8%), bone defects type II in 8 cases (42.1%) and bone defects type III in 4 cases (21.1%). In 9 cases (47.4%), reinfection of the revision endoprosthesis occurred, leading to a significant higher infection rate compared to cases without an additional nail (19.5%). No statistically significance between infection rate and extent of bone defect was found (*p* = .714). We integrated the spacer group and whether a spacer nail was used as variables of primary interest and controlling factors, such as age and diabetes (47% in patients with the additional use of nails, respectively 25% in patients without nails; *p* = .051) to account for intergroup differences in a Cox regression model (Table 5). Other variables did not show any statistical relevance and where therefore not integrated in the model. Based on the results there was an independent significant effect of a spacer nail on the hazard of reinfection (*p* = .005). The estimated hazard ratio for a spacer nail is 3.9, which indicates that the hazard of reinfection increases by a factor of 3.9 compared to situations without the use of spacer nails. This effect was independent from age, diabetes, and the proposed spacer groups.

**Table 5:** Cox proportional hazards regression of spacer survival without infection

**Variable**	**Haz. ratio**	**(*SE*)**	***z***	***p* Value**	**95% CI**
SVC	1.1	(0.69)	0.08	.938	(0.29; 3.8)
Spacer nail	3.9	(1.9)	2.8	.005	(1.5; 10)
Age	0.98	(0.02)	–0.89	.372	(0.94; 1.0)
Diabetes	0.80	(0.39)	–0.46	.643	(0.32; 2.0)

Abbreviations: Haz, hazard; SVC, superficial vancomycin coating.

## 4 DISCUSSION

The aim of our study was to investigate the risk of reinfection after septic revision endoprosthesis in two‐ or multiple staged procedures, using SVC spacer during first stage. We hypothesized that treatment of spacers with SVC will be of good compatibility and result in lower reinfection rates after reimplantation than conventional spacers. We observed a trend towards a lower prevalence of reinfections when SVC spacers were used, however, this finding remained without any statistically significance. Our secondary aim was to determine demographic and treatment factors associated with reinfection rates. Our data showed, that using a nail for additional stabilization of the static spacer leaded to a significant higher risk of reinfection after reimplantation of a revision endoprosthesis.

Persistent spacer infections in the SVC group (8.3%) were similar to the conventional spacer (6.9%), without a statistically significant risk reduction. In total, seven persistent spacer infections were diagnosed by histological and microbiological analysis from specimens, taken intraoperatively at the second stage, showing no significant higher risk of reinfection of the revision endoprosthesis during the follow‐up time. Only one of these cases had an occurrence of reinfection. Olsen et al.^24^ showed similar results in 2018, by using sonication cultures of the explanted spacer. In contrast to this findings, previous studies have shown that positive cultures during the second stage are associated with a higher failure rate.^25^, ^26^ Therefore, if there are evidently signs of persistent spacer infection preoperatively or intraoperatively, reimplantation of an implant seems precarious and spacer exchange should be performed.^27^

Nelson et al.^25^ used sonication of the spacer to identify persistent spacer infection. Overall, 50% of all patients, who showed positive microbiological findings in the sonication fluid, suffered from reinfection. In our study population, sonication of the explanted spacer was not done standardized during every second stage procedure, which is also recommended by Olsen et al.^24^ Kummer et al.^28^ concluded that elution of antibiotics from cement spacer along with the effect of sonication could inhibit bacterial growth, resulting in false negative results.

To detect potential complications of SVC, we investigated local and systemic vancomycin levels postoperatively, the clinical spacer performance, and creatinine levels. In contrast to high vancomycin concentrations locally, the serum levels did not increase over the observation period of five postoperative days. In fact, the maximum measured systemic vancomycin concentration of 10.3 μg/ml (median 2.2 μg/ml) is much lower than the recommended vancomycin through concentrations of 15–20 μg/ml for systemic therapy.^21^, ^29^ Consequently, only in one case (4.8%) a creatinine increase above 0.5 mg/dl was observed. In contrast, a creatinine increase above 0.5 mg/dl was measured in four cases (5.6%) in the conventional group. Thus, in our opinion, addition of superficial vancomycin coating is a safe method to enhance local antibiotics without the fear of systemically side effects, such as nephrotoxicity.

Different factors may affect the elution characteristics of PMMA bone cement and should be considered. Fist, the type and preparation method of the ALBC, second, the amount and type of antibiotics and third, the surface characteristics of the cement, as the majority of antibiotics elutes from the surface, related to the porosity and the cracks within the bone cement.^16^, ^19^, ^30^, ^31^ The mechanical effect of antibiotics on cement spacers is still a matter of debate. Other studies reported a huge amount of vancomycin (4–10 g per 40 grams bone cement) per spacer, with local vancomycin concentrations^32^, ^33^, ^34^, ^35^ comparable to our results. Using high amounts of antibiotics in bone cement influences the strength, which may lead to spacer failure. In a systemic review from 2014, Pivec et al.^36^ reported on spacer complications like loosening, dislocation, and fracture in static as well as articulating spacers from 2% to 14%. Similar results were observed by Struelens et al.,^37^ with major complications, such as dislocation (3%), fracture (5%), and subluxation (4%). Comparable complications were found in our cohorts, with no statistically significant difference between the SVC group and the conventional group. The results may indicate that SVC does not introduce a higher risk for mechanical failure than conventional cement spacers.

In 24 cases (25%) recurrent infection of the prosthesis was diagnosed after median 1.7 ± 4.0 years, showing a comparable reinfection rate than previous published.^6^, ^38^, ^39^, ^40^ Nevertheless, Gomez et. al. have stated in their work, that the success rates of a two stage exchange in different studies have been overestimated due to the fact that only the reinfection rate after reimplantation was analyzed, without evaluating the overall success or failure rate.^41^ They suggested to additionally include all failures between the first and the second‐stage, as well as cases where reimplantation did not take place.^41^ Considering all these cases in our study (spacer exchange = 26 cases, no reimplantation = 8 cases, amputation = 1 case, and prosthetic reinfections = 24 cases), we observed an overall failure rate of 45% after infection related surgical exchange procedures.

The implantation of SVC spacers did show a trend towards a lower prevalence of reinfections. However, this finding was not statistically significant. Due to the fact that SVC is used in clinical practice since May 2013 follow‐up observations were significantly shorter in the SVC group. As a consequence, cases of failure carry higher weights in survival analysis, which decrease the power of finding a statistical significance.

For additional stabilization, especially in cases of massive bone loss around the knee joint, nails may be used together with PMMA bone cement spacer.^42^, ^43^, ^44^, ^45^ In our cohort reinfection of the revision endoprosthesis occurred significantly more often in cases where additional nails were used during the spacer period, although, nails were also used in cases with bone defects type I or II (15 cases) and not only in cases with massive bone loss (4 cases). One reason might be the biofilm formation around the inserted metal, which protects the microbes from antibiotics and host immune responses during the spacer interval.^46^ Second, nails additionally with spacers are hardly used in patient with multiple revision, leading to extensive damage of periarticular bone and soft tissues and therefore, used as the last option before amputation.^47^, ^48^

The identification of a pathogen was only possible in 45% of cases. This rather low number is explained by the long study period starting in 2005. Systematic improvement of diagnostic sensitivity (e.g., sonication of explanted material, at least five tissue cultures) were implemented in 2016.

This study has some limitations. First, the retrospective cohort study model reduces the level of evidence. Second, as SVC spacers are a rather new approach, follow‐up observations and group balances are yet limited. To increase the power of detecting a statistical significance, multicenter, randomized controlled trials would be preferable for further investigations. Third, a very high rate of culture negative cases resulted due to poor intraoperative specimen collection in the past. Interpretations of culture specific treatment outcomes were therefore not available in the current study. Newer guidelines have been defined to increase the detection rate and may enable studies on local antibiotic specific targeting in future studies.

## 5 CONCLUSION

In our retrospective cohort study, superficial vancomycin coating of spacers in a two‐ or multiple stage‐exchange did achieve high local vancomycin concentrations without systemically side effects. A trend towards a lower prevalence of revision prosthetic reinfection with SVC spacers was observed but without statistical significance. Our data showed, that the rate of endoprosthesis reinfection was significantly higher when nails were used in combination with PMMA bone cement spacers.

## AUTHOR CONTRIBUTIONS

Florian Amerstorfer, Martina Schober, and Mathias Glehr substantial contributed to research design. Florian Amerstorfer, Stefan Fischerauer, Sebastian Klim, and Martina Schober analyzed and interpreted the data. Florian Amerstorfer, Thomas Valentin, Andreas Leithner, Mathias Glehr drafted the paper and revised it critically. All authors have read and approved the final submitted manuscript.
